# Blockade of Inflammatory Markers Attenuates Cardiac Remodeling and Fibrosis in Rats with Supravalvular Aortic Stenosis

**DOI:** 10.3390/biomedicines11123219

**Published:** 2023-12-05

**Authors:** Francine Duchatsch, Danyelle S. Miotto, Lidieli P. Tardelli, Thiago J. Dionísio, Dijon S. Campos, Carlos F. Santos, Katashi Okoshi, Sandra L. Amaral

**Affiliations:** 1 Joint Graduate Program in Physiological Sciences, PIPGCF UFSCar/UNESP, Rodovia Washington Luiz, km 235 Monjolinho, 676, São Carlos 13565-905, SP, Brazil; francine.duchatsch@gmail.com (F.D.); dany.miotto21@gmail.com (D.S.M.); lidielitardelli@gmail.com (L.P.T.); 2 Department of Biological Sciences, Bauru School of Dentistry, USP—University of São Paulo, Alameda Octávio Pinheiro Brisolla, 9–75, Bauru 17012-901, SP, Brazil; thiagoj@usp.br (T.J.D.); cfsantos@fob.usp.br (C.F.S.); 3 Department of Internal Medicine, Botucatu Medical School, São Paulo State University (UNESP), Av. Prof. Mário Rubens Guimarães Montenegro, s/n, Botucatu 18618-687, SP, Brazil; dijon.campos@unesp.br (D.S.C.); katashi.okoshi@unesp.br (K.O.); 4 Department of Physical Education, School of Sciences, São Paulo State University (UNESP), Av. Eng. Luiz Edmundo Carrijo Coube, 14-01—Vargem Limpa, Bauru 17033-360, SP, Brazil

**Keywords:** cardiac remodeling, left ventricle, echocardiogram, glucocorticoids, inflammatory cytokines

## Abstract

Since cardiac inflammation has been considered an important mechanism involved in heart failure, an anti-inflammatory treatment could control cardiac inflammation and mitigate the worsening of cardiac remodeling. This study evaluated the effects of dexamethasone (DEX) and ramipril treatment on inflammation and cardiac fibrosis in an experimental model of heart failure induced by supravalvular aortic stenosis. Wistar rats (21d) were submitted to an aortic stenosis (AS) protocol. After 21 weeks, an echocardiogram and a maximal exercise test were performed, and after 24 weeks, rats were treated with DEX, ramipril or saline for 14d. The left ventricle (LV) was removed for histological and inflammatory marker analyses. The AS group showed exercise intolerance (−32% vs. Sham), higher relative wall thickness (+63%), collagen deposition and capillary rarefaction, followed by cardiac disfunction. Both treatments were effective in reducing cardiac inflammation, but only DEX attenuated the increased relative wall thickness (−17%) and only ramipril reduced LV fibrosis. In conclusion, both DEX and ramipril decreased cardiac inflammatory markers, which probably contributed to the reduced cardiac fibrosis and relative wall thickness; however, treated AS rats did not show any improvement in cardiac function. Despite the complex pharmacological treatment of heart failure, treatment with an anti-inflammatory could delay the patient’s poor prognosis.

## 1. Introduction

Cardiac inflammation has been considered an important mechanism involved in the development and evolution of chronic heart failure (CHF) [1,2], which represents a major public health burden. Under situations of pressure overload, such as aortic stenosis, the heart undergoes adaptations in its structure as an adaptive mechanism in the face of increased load. This structural adaptation is called cardiac remodeling and includes changes in the size, shape and function of the heart [3,4,5,6,7,8,9,10,11]. It is already known that inflammatory markers play a role in regulating cardiac structure and function, particularly in the progression of CHF, and various strategies to counterbalance different aspects of the inflammatory response should be encouraged. Although several inflammatory markers such as interleukin 1 (IL1) [1,12,13,14,15,16], interleukin 6 (IL-6) [1,17,18,19,20], tumor necrosis factor α (TNFα) [1,11,19,21,22,23,24] and transforming growth factor β (TGFβ) [25,26] have been shown in a failing heart, the inhibition of these cytokines it still controversial. While some authors have shown improved cardiac remodeling [13,21,27], which could contribute to reduce cardiac dysfunction [3,25,28] and thus improve the prognosis of the CHF, others have shown no significant improvements [23]. 

Dexamethasone (DEX), as well as other glucocorticoids, is known for its potent anti-inflammatory effects, but there are some controversial results regarding its effects on cardiac remodeling and function [27,29,30,31,32,33,34]. It has been shown that treatment with DEX is able to inhibit inflammatory cytokines, myocardial fibrosis and reactive oxygen species (ROS), resulting in an improvement in cardiac remodeling and cardiac function in an experimental protocol of CHF in rats induced by left coronary artery ligation [27,31,33,34]. In contrast, another study demonstrated that DEX treatment inhibited the regeneration and recovery of cardiac function in the heart of 2-day-old post-myocardium infarction pigs [32]. Although there is still no clinical indication of glucocorticoids for the treatment of CHF [35], the attention for the cardioprotective effect of the glucocorticoids is growing [36,37,38]. Recently, some studies have shown that the use of glucocorticoids may be safe, effective and feasible to treat patients with CHF and impaired ejection fraction [36] and patients with chronic inflammatory cardiomyopathy [37,38]. However, almost nothing is known about the effects of DEX treatment on cardiac inflammation and cardiac remodeling induced by aortic stenosis. 

Because myocardial inflammation, hypertrophy and fibrosis can be induced by overactivation of the renin-angiotensin system aldosterone [39,40], the guideline-directed medical therapy for HF includes the angiotensin-converting enzyme inhibitors, especially for those patients with a reduced ejection fraction [35]. Therefore, several studies are suggesting the potent role of ramipril to treat cardiac patients with infarction and aortic stenosis [40,41,42,43,44]. However, more studies are needed to better understand the relationship between the renin-angiotensin system, inflammation, cardiac remodeling and aortic stenosis. Thus, this study aimed to evaluate the effects of DEX and ramipril treatment on inflammation and cardiac fibrosis in an experimental model of CHF induced by supravalvular aortic stenosis and their influence on cardiac remodeling. We proposed the following hypothesis: DEX and ramipril alleviate cardiac remodeling through their anti-inflammatory effects.

## 2. Materials and Methods

### 2.1. Animal Care

All experimental protocols were approved by the Committee for Ethical Use of Animals (CEUA) of the School of Sciences, São Paulo State University, UNESP, campus of Bauru, SP (#1044/2019 Vol. 1). 

Forty-three male Wistar rats 60–80 g (21 days old) were obtained from the animal breeding facility at São Paulo State University (UNESP, campus of Botucatu, SP) and were housed in the Animal Facility Maintenance at the School of Sciences, São Paulo State University (UNESP, campus of Bauru, SP) with water and food (Biobase, Águas Frias, SC, Brazil) ad libitum. Rats were housed in a temperature-controlled room (22 ± 2 °C) and kept on a 12:12 h light/dark cycle. 

### 2.2. Aortic Stenosis Surgery

The supravalvular aortic stenosis (AS) surgery was performed in 21-day-old rats (60–80 g). Thirty-one rats were anesthetized with ketamine hydrochloride (50 mg/kg, intraperitoneal, i.p.) and xylazine hydrochloride (10 mg/kg, i.p.). Subsequently, the trichotomy of the anterior region of the thorax of the rats was performed, and then, they underwent a median thoracotomy. After, the ascending aorta was dissected and a stainless-steel clip of 0.6 mm of internal diameter was placed approximately 3 mm from its root. The rats received saline intraperitoneally (1 mL) and, during surgery, were manually ventilated with 100% oxygen positive pressure [1,2]. The other twelve rats were submitted to a sham surgery, in which all surgical procedures were performed, except for the placement of the stainless-steel clip. According to other studies, eighteen weeks after AS surgery would be enough to induce cardiac disfunction [3]. Thus, as the aim of this study was to investigate cardiac dysfunction in severe heart failure, we waited 24 weeks after surgery to start the DEX and ramipril treatment protocol. Body weight was measured weekly for months and daily during treatment periods.

### 2.3. Maximal Exercise Capacity Test

**Figure 1:**
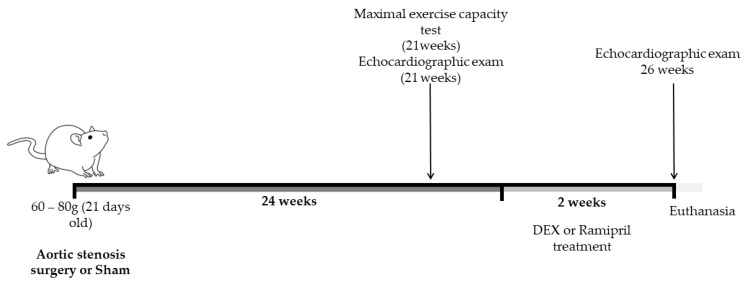
Experimental design. Wistar rats were submitted to an aortic stenosis (AS) protocol or sham surgery, and 21 weeks after surgery, all rats were submitted to a physical capacity test and transthoracic echocardiography for division of the groups. After 24 weeks of surgery, animals were treated with DEX (50 μg/kg/day, s.c.), ramipril (10 mg/kg/day, gavage) or saline for 14 days. After the experimental protocol, the animals were submitted to an echocardiographic examination and euthanasia.

As shown in Figure 1, after 21 weeks of surgery, a physical capacity test was performed to investigate the effects of the chronic AS procedure on the physical capacity of the animals. After 1 week of adaptation on a motorized treadmill (5 m/min/day), all rats were subjected to a maximal exercise capacity test that consisted of running on a treadmill at 6 m/min, which was increased by 3 m/min every 3 min until exhaustion, with 0% grade elevation until the animal stopped running spontaneously [4].

### 2.4. Echocardiography Assessment

All rats underwent transthoracic echocardiography evaluation 21 weeks after AS surgery to homogenize the groups according to disease evolution and 26 weeks after surgery, on the 13th day of drug treatment, for treatment evaluation. The rats were anesthetized with ketamine hydrochloride (50 mg/kg, i.p.) and xylazine hydrochloride (1 mg/kg, i.p.). Echocardiography was performed with a 5–11.5 MHz multifrequency probe (General Electric Medical Systems, Vivid S6, Tirat Carmel, Israel), as previously described [5]. The following variables were measured: heart rate, left ventricular (LV) diastolic diameter, left ventricular systolic diameter, LV posterior wall thickness, LV septal wall thickness, left atrial diameter, aorta diameter, relative wall thickness, LV mass, LV mass index, endocardial fractional shortening, midwall fractional shortening, posterior wall shortening velocity, ejection fractional (EF), Tei index, mitral E wave, mitral A wave, early (E)-to-late (A) diastolic mitral inflow and isovolumetric relaxation time.

### 2.5. Experimental Groups

Four groups were distributed as follows: (1) sham treated with saline (S = 12), sham surgery and received saline (s.c.) during the last 14 days; (2) aortic stenosis treated with saline (AS = 9), aortic stenosis surgery and received saline (s.c.) during the last 14 days; (3) aortic stenosis treated with DEX (ASD = 13), aortic stenosis surgery and received DEX (Decadron^®^; 50 µg/kg per day, at 9 a.m., s.c.) during the last 14 days; and (4) aortic stenosis treated with ramipril (ASR = 9), aortic stenosis surgery and during the last 14 days received ramipril (10 mg/kg per day, at 9 a.m., via gavage). Figure 1 illustrates the experimental design of the experimental protocol. 

### 2.6. Characterization of Heart Failure and Tissue Harvesting

At the end of experimental protocol, the groups were euthanized under anesthesia overload (xylazine hydrochloride 20 mg/kg and ketamine hydrochloride 160 mg/kg, i.p.). Before anesthesia, the presence of tachypnea was investigated, and after anesthesia, ascites, pleural effusion, atrial thrombi and liver congestion were investigated to observe clinical HF features [2]. After euthanasia, to evaluate cardiac hypertrophy, heart, atriums, left (LV) and right (RV) ventricles were removed, separated and weighed. Part of the left ventricle was removed for analysis of inflammatory markers and stored in a freezer at −80 °C, and another part of LV was stored for histological analysis. Since DEX treatment normally changes BW, tibia bone length was used for tissue normalization, and adrenal glands were removed for confirmation of DEX treatment efficacy. 

### 2.7. Analysis of the Inflammatory Markers 

Part of the left ventricle was homogenized (IKA T18, Staufen, Germany) in RIPA solution (Cell Signaling Technology, Danvers, MA, USA) plus 0.1% protease inhibitor cocktail (Pic, Sigma Aldrich, SLM, St. Louis, MO, USA) and 1% phenylmethanesulfonylfluoride (PMSF, Sigma Life Science, St. Louis, MO, USA). Then, the samples were centrifuged, and supernatant was stored at −20 °C. Inflammatory markers were analyzed by immunoassay (MILLIPLEX^®^ EMD Millipore Corporation, Burlington, MA, USA). The following panels were used to verify the presence of inflammatory markers in the LV: kit RAT Immunology Rat Cytokine/Chemokine (Cat. No. RECYTMAG-65K) 27 (Cat. No. RECYMAG65K27PMX) 27 (Bulk Cat. No. RECYMAG65PMX27BK) and kit Multi-Species TGFβ—Singleplex (Cat. No. TGFBMAG-64K-01) (Bulk Cat. No. TGFBMAG-64K-01BK); all the procedures were performed according to the manufacturer’s protocol. All samples, quality control samples and standards were prepared as recommended following the MILLIPLEX^®^ manufacturer’s instructions, using assay kit protocols with supplied diluents. The assay plate was then analyzed with the Luminex^®^ instrument equipped with xPONENT^®^ and Multiplex Analyst 5.1 software. The results were obtained in pg/mL [6].

### 2.8. Histological Analysis

Another part of LV was fixed in 4% buffered paraformaldehyde solution for 24 h. After that, tissues were embedded in Paraplast (Sigma, St. Louis, MO, USA). Histological transverse sections were performed using a manual microtome (Microm HM 325 Microtome, Southeast Pathology Instrument Service, Charleston, SC, USA) with disposable blades and placed on slides. Sections of 5 μm thickness (hematoxylin–eosin staining, HE, Easy Path, SP) were used for capillary density and myocyte diameter, while 7 μm thickness (Picrosirius Red staining, Merck-Millipore, Burlington, MA, USA) were used for collagen analyses. All images were captured using Leica MC170 HD camera, coupled to the Leica DM4 B microscope (200 or 400× magnification). Analyses (off-line) were performed using ImageJ software, on blinded slides [7].

### 2.9. Statistical Analysis

Data normality was verified using the Shapiro–Wilk test followed by the Anderson–Darling test, D’Agostino and Pearson test and Kolmogorov–Smirnov test. The Student’s *t*-test was used to analyze two groups with normal distribution and the Mann–Whitney Rank Sum test was used for two non-parametric groups. Log-rank (Mantel–Cox) and Gehan–Breslow–Wilcoxon tests were used to perform and analyze the survival rate curve, and Chi square was used for heart failure signs contingency. To analyze the effect of treatment with DEX and ramipril, one-way analysis of variance (ANOVA) for parametric data and Kruskal–Wallis for non-parametric data were used. In the presence of interactions, Tukey or Dunnett’s post hoc were used (*p* < 0.05). Results are presented as mean ± standard error of the mean. SigmaStat 3.11 software and GraphPad Prism 8.0.1 were used for statistical analyses and graphs.

## 3. Results

### 3.1. Aortic Stenosis Surgery Promoted Changes in the Survival Curve, Exercise Tolerance and Cardiac Structure and Function

**Figure 2:**
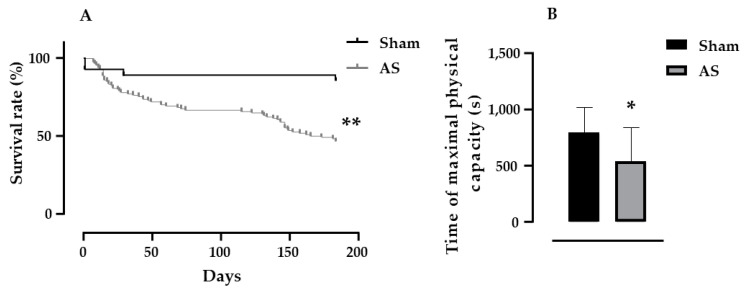
Black line (**A**) and black bar (**B**) represent the sham group, while gray line (**A**) and gray bar (**B**) represent aortic stenosis rats. Analysis of the survival rate (**A**) measured in both groups sham (*n* = 28) and aortic stenosis (AS, *n* = 113) and results of maximal physical capacity test (**B**) measured in both groups sham (*n* = 20) and aortic stenosis (AS, *n* = 23). Results are presented as mean ± standard error of the mean, significance: * *p* < 0.05, ** *p* < 0.01 vs. sham group.

The animals that were submitted to AS surgery (*n* = 113) had more deaths throughout disease development when compared to sham (*n* = 28) animals (Figure 2A, *p* = 0.0011). In addition, as expected, animals with AS were intolerant to exercise since they ran for 32% less time when compared with sham animals (Figure 2B, 537 ± 62 s vs. 791 ± 51 s, for AS vs. sham, *p* = 0.003). It is important to note that these results were obtained before both treatments (Figure 2). Exercise intolerance was one of the limiting symptoms present in HF, as well as other signs presented by the EA group, such as apathetic behavior, capillary changes, tachypnea, pleural effusion, atrial thrombi, ascites and liver congestion (*p* < 0.05, for AS vs. sham).

Table 1 shows that both groups had similar body weight increase (delta) during the 26 weeks after surgery (Δ = 341 ± 15 g, and 335 ± 14 g, for sham and AS, respectively). The hearts of AS animals were more hypertrophied than sham animals (+67%, *p* < 0.001), as well as the LV (+58%, *p* < 0.001), RV (+38%, *p* = 0.025) and atria (+188%, *p* < 0.001) vs. S. On the other hand, tibialis anterior muscle weight was reduced in animals with aortic stenosis (−14%, *p* = 0.025). Additionally, the AS group had an increase of 41% (*p* = 0.025) in the weight of the adrenal gland, when compared with the sham group.

The increase in heart structures in animals with AS was confirmed by the echocardiographic examination (Table 2) with a marked increase in the LV posterior wall thickness and LV septal wall thickness (+65%, *p* < 0.001), left atrial diameter (+44%, *p* < 0.001), left atrial diameter/aortic diameter (+46%, *p* < 0.001), relative wall thickness (+63%, *p* < 0.001), LV mass (+104%, *p* < 0.001) and LV mass index (+112%, *p* < 0.001), vs. the sham group. In addition, there was a reduction in the posterior wall shortening velocity (−18%, *p* = 0.009) and an increase in mitral E wave (+43%, *p* < 0.01) and early €-to-late (A) diastolic mitral inflow (+96%, *p* < 0.05) vs. the sham group.

**Figure 3:**
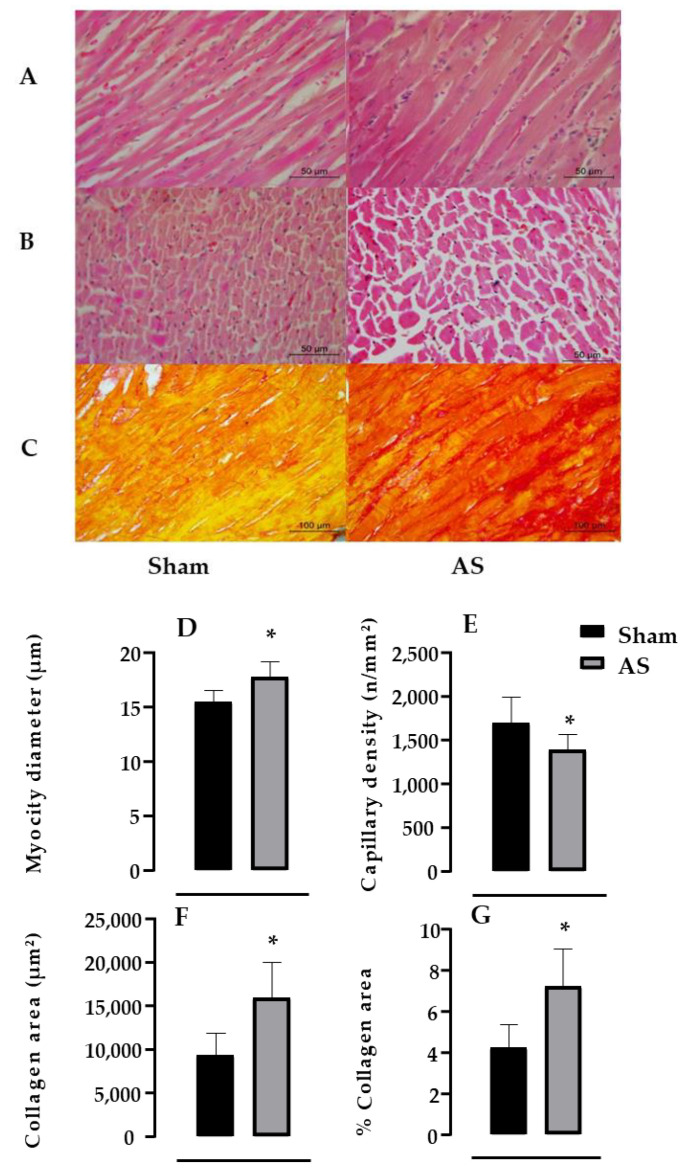
Upper panel: Representative images of hematoxylin and eosin (H and E, (**A**,**B**)) and Picrosirius Red (**C**) staining for each group; (**A**) longitudinal section showing the myocytes as demonstrated by the black line; (**B**) transversal section showing the capillaries as indicated by the black arrow; and (**C**) transversal sections showing the collagen deposition, in which collagen fibers can be seen in red and cardiac musculature in yellow. Bar: 50 µm, increase of 400× (**A**,**B**) and 100 µm, increase of 200× (**C**). Bottom panel: From hematoxylin–eosin technique, analyses of •myocyte diameter (µm, (**D**)) and •capillary density on the left ventricle (n/mm^2^, (**E**)). From picrosirius red staining images, densitometric results of left ventricle collagen deposition area (μm^2^, (**F**)) and % of collagen area (**G**) in all analyzed groups: sham (S, *n* = 12) and aortic stenosis (AS, *n* = 9). Results are presented as mean ± standard error of the mean. • means parametric test. Significance: * *p* < 0.05, vs. sham group.

Figure 3, upper panel, illustrates a cross section of the LV from one rat of each group (sham or AS group) stained with HE (A and B) and Picrosirius Red (C). The bottom panel shows the analysis of each group. It is shown that the AS group showed an increase in the myocyte diameter (A, +15%, *p* < 0.001) and collagen deposition (C, +70%, *p* < 0.001), accompanied by a reduction in the capillary density (D, −18%, *p* = 0.014) vs. the sham group.

**Figure 4:**
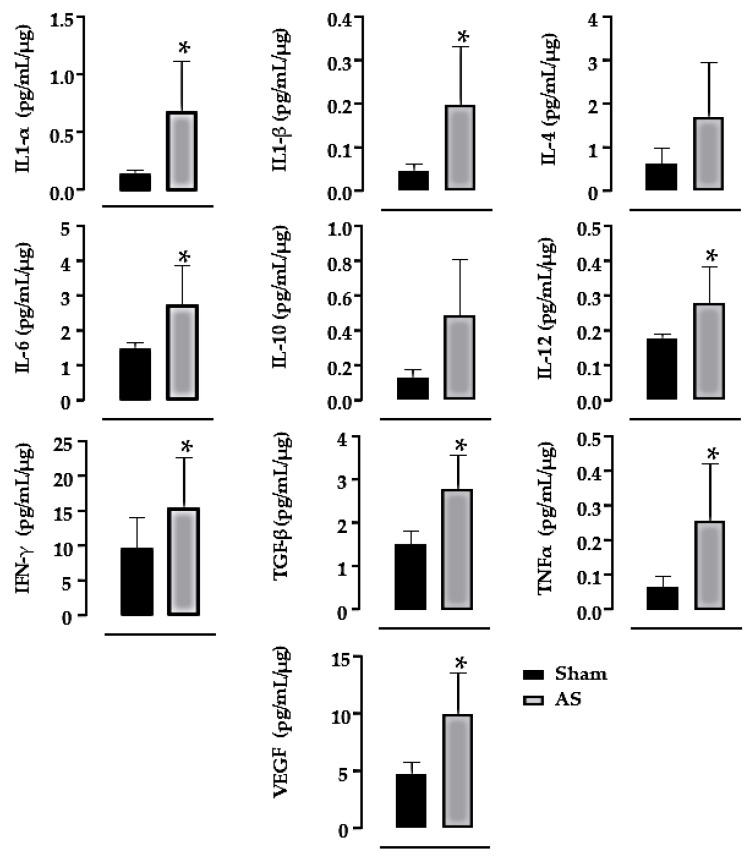
Analysis of cardiac inflammatory markers: interleukin 1α (IL1-α), interleukin 1 β (•IL1-β), interleukin 4 (•IL-4), interleukin 6 (IL-6), interleukin 10 (•IL-10), interleukin 12 (IL-12), interferon gama (IFN-γ), transforming growth factor β (TGF-β), tumor necrosis factor α (•TNF-α) and vascular endothelial growth factor (•VEGF). Sham (S, *n* = 10) and aortic stenosis (AS, *n* = 9). Results are presented as mean ± standard error of the mean. • means parametric test. Significance: * *p* < 0.05, vs. sham group.

The inflammatory profile, shown in Figure 4, shows that AS animals had an increase in the level of inflammatory markers such as interleukin 1α (IL1-α, +382%, *p* = 0.037), interleukin 1 β (IL1-β, +331%, *p* = 0.037), interleukin 6 (IL-6, +86%, *p* = 0.004), interleukin 12p70 (IL-12, +60%, *p* = 0.025), interferon gama (IFN-γ, +60%, *p* = 0.043), transforming growth factor β (TGFβ, +82%, *p* = 0.002), tumor necrosis factor α (TNFα, +281%, *p* = 0.020) and vascular endothelial growth factor (VEGF, +112%, *p* = 0.008) vs. the sham group.

### 3.2. DEX Treatment Attenuated Cardiac Hypertrophy While Ramipril Treatment Improves Collagen Deposition and Both Treatments Improve Cardiac Inflammatory Profile

All groups had a similar body weight increase during the 24 weeks after surgery but before any treatment (340 ± 14 g, 330 ± 17g and 324 ± 15 g, for AS, ASD and ASR, respectively). As shown in Table 3, the DEX treatment significantly reduced the BW of rats, when compared with the AS group (−588%, *p* < 0.001). The lung (w/d) was reduced with ramipril treatment when compared with the AS group (−12%, *p* = 0.0049) and the adrenal gland was reduced in both treatments vs. the AS group (−45% for ASD, *p* = 0.0002 and −26% for ASR, *p* = 0.0305), as shown in Table 3.

The echocardiographic data are shown in Table 4. It is possible to see that stenosis DEX-treated rats (ASD) presented lower values of echocardiographic structural data when compared with stenosis control rats, such as −19% on posterior wall thickness (*p* = 0.0172), −19% on interventricular septum (*p* = 0.0172) and −17% on relative wall thickness (*p* = 0.0043). Ramipril treatment did not change any of the echocardiographic data compared with the AS group.

**Figure 5:**
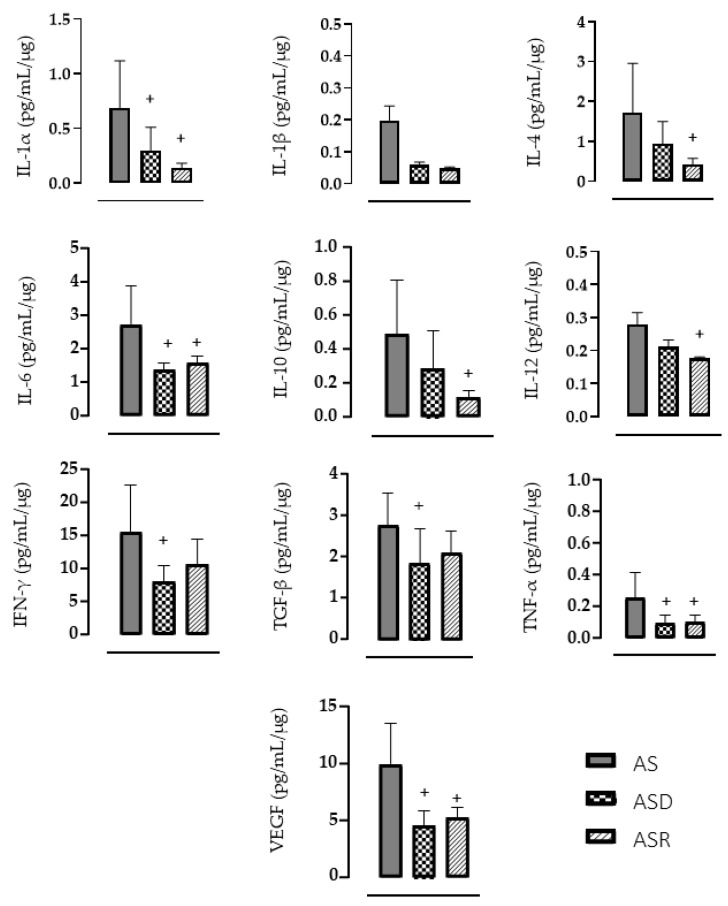
Analysis of cardiac inflammatory markers: interleukin 1α (IL1-α), interleukin 1 β (IL1-β), interleukin 4 (•IL-4), interleukin 6 (•IL-6), interleukin 10 (IL-10), interleukin 12 (IL-12), interferon gama (•IFN-γ), transforming growth factor β (TGFβ), tumor necrosis factor α (•TNFα) and vascular endothelial growth factor (•VEGF). Aortic stenosis (AS, *n* = 9), aortic stenosis treated with DEX (ASD, *n* = 9) and aortic stenosis treated with ramipril (ASR, *n* = 9). Results are presented as mean ± standard error of the mean. • means parametric test. Significance: ^+^ *p* < 0.05 vs. AS group.

On the other side, DEX treatment blocked the increase in inflammatory markers compared with the AS group. Figure 5 shows that the ASD group presented lower values of IL1-α (−57%, *p* = 0.0160), IL-6 (−50%, *p* = 0.0008), IFN-γ (−49%, *p* = 0.0064), TGFβ (−33%, *p* = 0.0269), TNFα (−61%, *p* = 0.0073) and VEGF (−54%, *p* < 0.0001) vs. AS. Similarly, ramipril treatment blocked the increase in inflammatory markers such as IL1-α (−79%, *p* = 0.0013), IL4 (−75%, *p* = 0.0060), IL-6 (−42%, *p* = 0.0046), IL10 (−76%, *p* = 0.0060), IL12 (−37%, *p* = 0.0398), TNFα (−60%, *p* = 0.0113) and VEGF (−47%, *p* = 0.0006) vs. the AS group.

**Figure 6:**
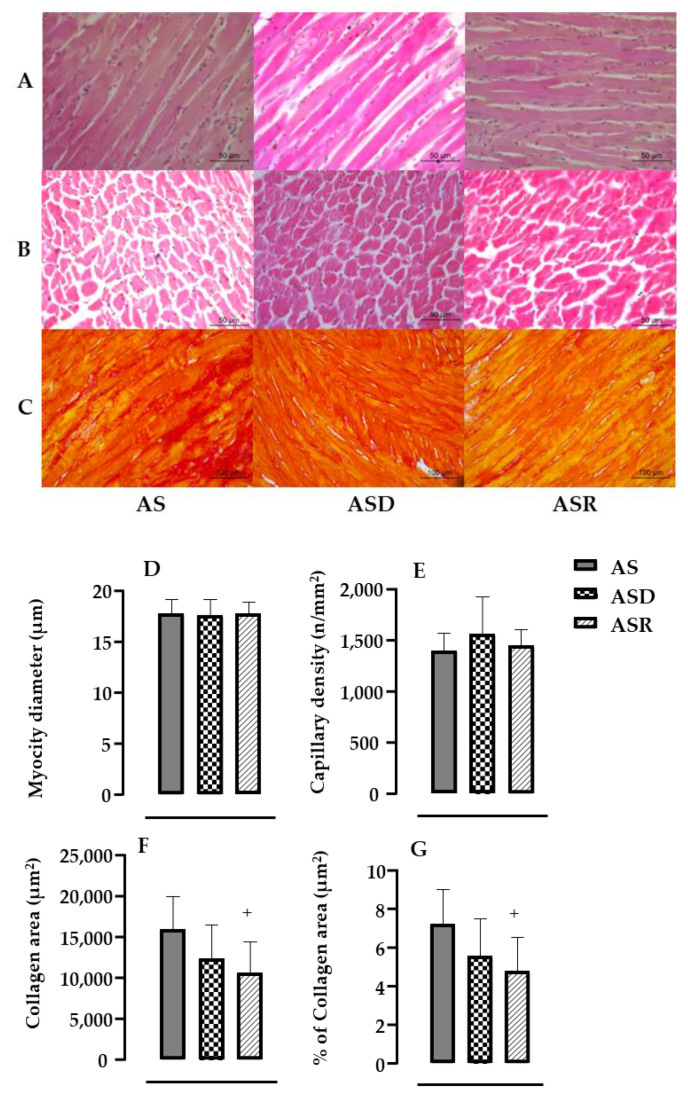
Upper panel: Representative images of hematoxylin and eosin (H and E, (**A**,**B**)) and Picrosirius Red (**C**) staining for each group; (**A**) longitudinal section showing the myocytes as demonstrated by the black line; (**B**) transversal section showing the capillaries as indicated by the black arrow; and (**C**) transversal sections showing the collagen deposition, in which collagen fibers can be seen in red and cardiac musculature in yellow. Bar: 50 µm/increase of 400× (**A**,**B**) and 100 µm/ increase of 200× (**C**). Bottom panel: From hematoxylin–eosin technique, analyses of •myocyte diameter (µm, (**D**)) and capillary density on the left ventricle (n/mm^2^, (**E**)). From picrosirius red staining images, densitometric results of left ventricle collagen deposition area (μm^2^, (**F**)) and % of collagen area (**G**) in all analyzed groups: aortic stenosis (AS, *n* = 9), aortic stenosis treated with DEX (ASD, *n* = 9) and aortic stenosis treated with ramipril (ASR, *n* = 9). Results are presented as mean ± standard error of the mean. • means parametric test. Significance: ^+^ *p* < 0.05 vs. AS group.

Figure 6, top panel, illustrates a cross-section of the LV from one rat from each group stained with HE (A and B) and Picrosirius Red (C). The bottom panel shows the densitometric analysis in all groups. There was no significant change in the myocyte diameter or capillary density after treatments. However, rats with aortic stenosis treated with ramipril showed a decrease in collagen deposition (−34%, *p* = 0.0238) compared with the AS group.

## 4. Discussion

The main results of this study were that chronic heart failure, induced by supravalvular aortic stenosis, was characterized by left ventricular hypertrophy, fibrosis, myocardial inflammation and systolic and diastolic dysfunction, however, with a preserved ejection fraction. Both DEX and ramipril treatments improved myocardial inflammation, but only DEX treatment improved cardiac hypertrophy, whereas ramipril attenuated cardiac fibrosis. 

Chronic heart failure is a complex syndrome defined as an inability of the heart to maintain sufficient tissue perfusion, with a high mortality and hospitalization rate [45]. In addition, patients with CHF have associated comorbidities, complex pharmacological treatment and limiting symptoms such as fluid retention, shortness of breath, fatigue and intolerance to effort, which corresponds to a poor quality of life [45]. Studies have shown that supravalvular aortic stenosis surgery is an experimental model of HF because it causes almost all the signs and symptoms of HF [7,10,11]. Therefore, in the first part of this study, we aimed to confirm and present a brief characterization of the rats submitted to surgery for supravalvular aortic stenosis.

The present study revealed that the AS group had almost all of the HF clinical signs, such as tachypnea, ascites, pleural effusion, atrial thrombi and liver congestion, which probably contributed to decreasing the time on the treadmill during the maximal physical capacity test, when compared with the sham group, suggesting an intolerance to physical exercise, which is an important consequence of HF, in accordance with another study [11]. In consequence, it was observed that the AS group had a lower survival rate, compared with the sham group. It is important to note that the number of rats present in this study is relative to the survivors. Although these animals presented reduced tibialis anterior muscle mass, suggesting muscle atrophy, they did not have a significant reduction in their BW, which agrees with other studies [4,5,8,10,46]. 

There are several etiologies of CHF, since it is the outcome of many cardiovascular diseases, so several pathological conditions can lead to the development of this syndrome, such as coronary artery disease, hypertension, valvular diseases, arrhythmia, cardiomyopathy, congenital diseases, infectious diseases, drug-induced diseases, infiltrative diseases, pericardial diseases, endomyocardial diseases, storage disorders, metabolic diseases and neuromuscular diseases [47], and this pathological heterogeneity makes treatment a challenge [1]. All organs may suffer from this syndrome but, at first, the heart undergoes some adjustments in its structure to deal with the imposed overload [1,2]. Therefore, the presence of cardiac hypertrophy is also a well-known feature of CHF, for maintaining adequate blood perfusion [3,4,5,6,7,8,9,10,11]. Despite the presence of cardiac hypertrophy, heart failure can be classified in humans as a preserved ejection fraction (≥50%), a slightly reduced ejection fraction (41–49%) and a reduced ejection fraction (≤40%) [35,42].

In agreement, the results of this present study revealed that 26 weeks after supravalvular AS surgery, the rats showed an increase in the heart, LV and atrium muscle weight. Through the echocardiographic examination, it was possible to observe an increase in the diastolic thickness of the posterior wall and LV interventricular septum, as well as an increase in the relative thickness of the LV, an increase in the LV mass and LV mass index. In addition, there was an increase in the LA, which agrees with other studies [3,4,5,6,7,8,9,10,11]. The increased thickness of the LV in this present study was confirmed by the higher myocyte diameter, present in the AS group and evaluated by histology. This response occurs due to the placement of the clip around the ascending aorta, which determines a decrease in the aortic lumen, and this reduction is a pathological stimulus that causes pressure overload. This increase in pressure overload promotes an increase in the cardiac mass, resulting in concentric hypertrophy, to normalize the increase in the systolic wall stress and allow for an improvement in cardiac function [48]. However, the LV hypertrophy induced by aortic stenosis in this present work worsened the cardiac function of these animals, shown by a reduction in the posterior wall shortening velocity, which indicates a reduction in systolic function [36]. The increases in the left aortic diameter, left aortic diameter/aortic diameter, mitral E wave and mitral E/A wave observed in AS rats suggest diastolic dysfunction. Furthermore, the Tei index was higher in the AS group (even though not significant), which indicates a worsening of myocardial performance. Despite this, the ejection fraction was still preserved in these rats.

There are several mechanisms implicated in cardiac remodeling, which includes cardiac inflammation [11,25]. It has been shown that cardiac inflammation is initiated after a cardiac insult, which induces cardiac hypertrophy, apoptosis, matrix protein degradation and myocardial collagen deposition, which indeed may contribute to a decrease in cardiac function [1]. In agreement, the AS group of this present study showed an increase in the cardiac levels of pro- and anti-inflammatory markers, such as IL1-α, IL1-β, IL-4, IL-6, IL-10, IL-12p70, IFN-γ, TGFβ, TNFα and VEGF, which agrees with other studies [11,19,20,24,25,26]. This happens because after myocardial injury, inflammatory markers are released with the objective of tissue repair and adaptation after the injury [49]. Cardiac hypertrophy occurs during the process of tissue repair and adaptation, in which some inflammatory markers can directly influence the increase in cardiac mass, for instance, TNFα binds to its receptor 1 and induces cardiac hypertrophy [24]. Another study shows that activation of mitogen-activated protein kinase and Ca^2+^/calmodulin-dependent protein kinase II- signal transducers and activators of transcription signaling pathways 3 by IL-6 also induce cardiac hypertrophy after a cardiac insult [20]. It is important to point out that some cytokines can also stimulate the secretion of other cytokines and inflammatory markers, such as IL1, which stimulates the secretion of IL-6 [50]. It has been demonstrated that the increased release of inflammatory cytokines can also induce fibrosis [14,51], which is in agreement with the results of the present study, which identified a greater deposition of collagen on the myocardium. Furthermore, the LV capillary density was reduced in these rats with AS, which may also have contributed to impaired myocardial contractility.

The second part of this study aimed to evaluate the effects of two different drugs, known for their anti-inflammatory role, on myocardial inflammation and fibrosis and their influence on cardiac remodeling in rats undergoing induction of supravalvular aortic stenosis. 

Although several adverse effects have been associated with the use of glucocorticoids, such as facial and leg swelling, weight gain and hypertension, some studies have suggested that glucocorticoids may be safe, effective and feasible for treating patients with CHF and an impaired ejection fraction [52], as well as patients with acute or chronic inflammatory cardiomyopathy [37,38]. Likewise, while there are some studies demonstrating that DEX treatment induces hypertrophy in healthy animals [30,53,54], the low-dose of DEX treatment, used in this present study, per se usually does not change any structural parameter, as shown in previous results published by our group using Wistar healthy rats [29,55]. In fact, recently, our group demonstrated that treatment with a low dose of DEX (same as in this present study) improved cardiac remodeling, through attenuation of LV hypertrophy, myocardial collagen deposition and stimulation of myocardial capillary angiogenesis, followed by a small but significant improvement in LV systolic and diastolic function [29] in spontaneously hypertensive rats (SHR). However, almost nothing was known about the effects of DEX treatment on the heart of AS-induced heart failure.

Differently from DEX, angiotensin converting enzyme inhibitors are highly recommended to reduce morbidity and mortality in patients with HF, according to the Guideline for the Management of Heart Failure [35]. In agreement, a recent systematic review and meta-analysis has pointed out that renin angiotensin system inhibitor treatment, including the angiotensin converting enzyme inhibitor, significantly reduced short- and long-term mortality in AS patients who underwent aortic valvar replacement, but it was also safe for patients with AS who did not undergo valve replacement and did not increase mortality [42]. 

Although this study did not investigate the exact inhibitory mechanisms of DEX and ramipril on the supravalvular aortic stenosis myocardial cytokine generation, our results confirm their significant anti-inflammatory effect, as shown by other studies [27,33,34,43,44,56]. The present study showed that myocardial levels of TNFα, IL1-α and IL-6 were lower after both treatments, DEX and ramipril compared to AS rats. On the other hand, TGFβ and IFN-γ levels were lower only after DEX treatment and IL-4, IL-10, IL-12p70 and VEGF levels were reduced only after ramipril treatment. Myocardial IL1-β level was not significantly reduced by any of the treatments.

Interestingly, structural echocardiographic parameters, such as posterior wall thickness, septal wall thickness and relative wall thickness, but not LV mass or LV mass index, observed in the first part of this study as being higher in AS rats, were attenuated only after DEX treatment. Even though TNFα and IL-6 were also lower in the ramipril group, the lower LV hypertrophy observed in the DEX group may be related to the lower levels of the pro-inflammatory cytokines TNFα, IL1-α and IL-6, TGFβ and IFN-γ, since the association of these cytokines with myocardial hypertrophy has been shown [24,51,57]. Although new evidence has shown the anti-inflammatory and anti-hypertrophic role of TNFα, through its receptor 2 [24], the TNF receptor 1 is highly expressed in the failing heart after AS, and several studies have demonstrated reduced cardiac inflammation through lower levels of TNFα. 

As cardiac wall thickness (posterior wall thickness, septal wall thickness and relative wall thickness) was reduced after DEX treatment, while the myocyte diameter remained increased (due to the experimental model of AS), we evaluated myocardial collagen deposition, which is commonly induced by inflammatory cytokines [14,51]. Actually, myocardial fibrosis occurs as part of the hypertrophic response and has been considered an independent risk of mortality in aortic stenosis [35]. Inflammatory markers, such as TNF-α and IL-1, are capable of activating metalloproteinases that are responsible for collagen degradation and synthesis [58,59]. In addition, it has been shown that TGFβ upregulates collagen synthesis through a TGFβ/TGFR/smad2-3 pathway [60]. The histological analysis of this present study revealed that DEX slightly reduced the myocardium collagen deposition. Although this answer was not statistically significant in this present study, we have confirmed this effect in another study using SHR [29]. Even though treatment with ramipril, in this present study, did not improve cardiac hypertrophy, it significantly reduced cardiac fibrosis. Even though ramipril did not significantly reduce the TGFβ protein level, which is an important fibrotic factor, it has been shown that angiotensin II activates the synthesis and secretion of TGF-beta1 and that the angiotensin converting enzyme and TGF-beta1 mRNA are associated in the human heart with AS [40]. Therefore, we may speculate that the small reduction in the myocardial TGFβ level, associated with a lower level of IL-6, could be contributing to the ramipril-induced lower cardiac fibrosis in AS rats. 

While DEX and ramipril decreased the cardiac inflammatory markers, which probably contributed to reducing cardiac fibrosis and relative wall thickness, AS rats did not show any improvement in cardiac function. Therefore, our hypothesis was partially confirmed, since DEX and ramipril decreased most of the cardiac inflammatory markers and fibrosis, but they did not improve cardiac function. Maybe the duration of the treatment was not enough, and therefore, a longer period of treatment could reach better responses. 

Taken together all the information presented in this study, chronic heart failure, induced by supravalvular aortic stenosis, was characterized by left ventricular hypertrophy and fibrosis, myocardial inflammation, and systolic and diastolic dysfunction. Although this experimental model presented a preserved ejection fraction, it was characterized by exercise intolerance and several heart failure limiting symptoms such as apathetic behavior, capillary changes, tachypnea, pleural effusion, atrial thrombi, ascites and liver congestion, which probably contributed to the high mortality rate observed in this present study. While some recent studies have suggested that glucocorticoids may be safe, effective, and feasible for treating patients with heart failure with an impaired ejection fraction, the novelty of this present study is that DEX treatment improved myocardial inflammation and cardiac hypertrophy in rats with a preserved ejection fraction and did not cause any other important adverse effects. Likewise, ramipril treatment also reduced myocardial inflammation and attenuated cardiac fibrosis. Nevertheless, none of the treatments mitigated the reduced systolic and diastolic function observed in those rats with supravalvular aortic stenosis. We cannot avoid considering that the results could be better if we had used a model of aortic stenosis not induced by a stainless-steel clip around the aorta. Also, we believe that longer periods of treatment with ramipril could induce a stronger reduction in myocardial inflammation and attenuate cardiac hypertrophy, which could contribute to improve rat prognosis. 

## 5. Conclusions

In conclusion, the present study suggests that DEX and ramipril may be beneficial in mitigating the evolution of heart disease induced by aortic stenosis due to a small attenuation of cardiac remodeling induced by lower myocardial inflammation. Therefore, despite the complex pharmacological treatment of heart failure, treatment with an anti-inflammatory such as DEX or ramipril could delay the patient’s poor prognosis.

## Figures and Tables

**Table 1:** Morphological data.

	S (*n* = 12)	AS (*n* = 9)
Delta body weight (g)—(26°–1° weeks)	341 ± 15	335 ± 14
Tibia bone length (cm)	4.217 ± 0.024	4.211 ± 0.035
Liver (w/d)	3.083 ± 0.054	2.903 ± 0.073
•Lung (w/d)	4.512 ± 0.169	4.763 ± 0.058
Heart/tibia (g/cm)	0.343 ± 0.027	0.573 ± 0.040 *
LV/tibia (g/cm)	0.216 ± 0.012	0.341 ± 0.015 *
RV/tibia (g/cm)	0.087 ± 0.013	0.119 ± 0.012 *
Atrium/tibia (g/cm)	0.033 ± 0.003	0.095 ± 0.018 *
Tibialis Anterior/tibia (g/cm)	0.189 ± 0.007	0.162 ± 0.009 *
•Adrenal gland/tibia (g/cm)	0.014 ± 0.001	0.020 ± 0.002 *

Delta body weight treatment period, tibia bone length, liver wet-to-dry weight ratio (w/d), lung wet-to-dry weight ratio (w/d), heart, left ventricle (LV), right ventricle (RV), atrium, tibialis anterior muscle and adrenal gland weight (sum of both adrenal gland) were normalized by tibia bone length in all groups: sham (S) and aortic stenosis (AS). Results are presented as mean ± standard error of the mean. • means parametric test. Significance: * *p* < 0.05, vs. sham group.

**Table 2:** Heart rate and echocardiographic data.

	S (*n* = 12)	AS (*n* = 9)
•HR (bpm)	277 ± 15	274 ± 5
LVDD (mm)	8.382 ± 0.305	8.464 ± 0.368
LVSD (mm)	4.368 ± 0.339	4.107 ± 0.423
PWT (mm)	1.313 ± 0.009	2.166 ± 0.102 *
SWT (mm)	1.314 ± 0.009	2.166 ± 0.102 *
LA (mm)	5.663 ± 0.164	8.144 ± 0.404 *
•LA/AO	1.343 ± 0.025	1.956 ± 0.099 *
RWT	0.316 ± 0.008	0.515 ± 0.024 *
LVM (g)	0.785 ± 0.058	1.601 ± 0.183 *
LVMI (g/kg)	1.667 ± 0.099	3.532 ± 0.332 *
•EFS %	48.479 ± 2.053	52.346 ± 2.847
•MFS %	28.780 ± 1.348	30.362 ± 1.476
•PWSV (mm/s)	39.899 ± 1.666	32.812 ± 1.692 *
EF %	0.856 ± 0.018	0.882 ± 0.022
•Tei index	0.484 ± 0.020	0.556 ± 0.042
Mitral E (cm/s)	74.636 ± 2.248	106.778 ± 11.289 *
Mitral A (cm/s)	45.545 ± 2.538	49.000 ± 8.949
E/A	1.686 ± 0.098	3.298 ± 0.819 *
•IVRT (ms)	27.250 ± 0.946	23.222 ± 2.165

Heart rate (HR), left ventricular (LV) diastolic diameter (LVDD), left ventricular systolic diameter (LVSD), LV posterior wall thickness (PWT), LV septal wall thickness (SWT), left atrial diameter (LA), aortic diameter (AO), relative wall thickness (RWT), LV mass (LVM), LV mass index (LVMI), endocardial fractional shortening (EFS), midwall fractional shortening (MSF), posterior wall shortening velocity (PWSV), ejection fractional (EF), mitral E inflow (mitral E), mitral A (mitral A) wave, early (E)-to-late (A) diastolic mitral inflow (E/A) and isovolumetric relaxation time (IVRT). Sham (S) and aortic stenosis (AS). Results are presented as mean ± standard error of the mean. • means parametric test. Significance: * *p* < 0.05, vs. sham group.

**Table 3:** Morphological data after dexamethasone and ramipril treatments.

	AS (*n* = 9)	ASD (*n* = 9)	ASR (*n* = 8)
Δ body weight (g) treatment period	−8 ± 3	−55 ± 4 ^+++^	−16 ± 4 ^#^
Tibia bone length (cm)	4.211 ± 0.035	4.189 ± 0.039	4.188 ± 0.030
Liver (w/d)	2.903 ± 0.073	2.975 ± 0.072	3.064 ± 0.114
Lung (w/d)	4.763 ± 0.058	4.432 ± 0.180	4.172 ± 0.176 ^++^
Heart/tibia (g/cm)	0.573 ± 0.040	0.491 ± 0.047	0.542 ± 0.050
•LV/tibia (g/cm)	0.341 ± 0.015	0.309 ± 0.014	0.345 ± 0.019
RV/tibia (g/cm)	0.119 ± 0.012	0.093 ± 0.015	0.103 ± 0.013
Atrium/tibia (g/cm)	0.095 ± 0.018	0.077 ± 0.018	0.098 ± 0.020
Tibialis Anterior/tibia (g/cm)	0.162 ± 0.009	0.144 ± 0.008	0.151 ± 0.005
•Adrenal gland/tibia (g/cm)	0.020 ± 0.002	0.011 ± 0.001 ^+++^	0.015 ± 0.001 ^+^

Delta body weight treatment period, tibia bone length, liver wet-to-dry weight ratio (w/d), lung wet-to-dry weight ratio (w/d), heart, left ventricle (LV), right ventricle (RV), atrium, tibialis anterior muscle and adrenal gland weight (sum of both adrenal gland) were normalized by tibia bone length: aortic stenosis (AS), aortic stenosis treated with DEX (ASD) and aortic stenosis treated with ramipril (ASR). Results are presented as mean ± standard error of the mean. • means parametric test. Significance: ^+^ *p* < 0.05, ^++^ *p* < 0.01, ^+++^ *p* < 0.001 vs. AS group and ^#^ *p* < 0.01 vs. ASD group.

**Table 4:** Heart rate value and echocardiographic data.

	AS (*n* = 9)	ASD (*n* = 9)	ASR (*n* = 8)
•HR (bpm)	274 ± 5	280 ± 11	262 ± 13
LVDD (mm)	8.464 ± 0.368	8.291 ± 0.378	7.775 ± 0.293
LVSD (mm)	4.107 ± 0.423	3.750 ± 0.550	4.043 ± 0.612
PWT (mm)	2.166 ± 0.102	1.758 ± 0.045 ^+^	2.234 ± 0.086
SWT (mm)	2.166 ± 0.102	1.758 ± 0.045 ^+^	2.234 ± 0.086
•LA (mm)	8.144 ± 0.404	7.753 ± 0.471	8.329 ± 0.684
•LA/AO	1.956 ± 0.099	1.889 ± 0.101	2.053 ± 0.162
RWT	0.515 ± 0.024	0.428 ± 0.013 ^+^	0.576 ± 0.016 ^#^
LVM (g)	1.601 ± 0.183	1.146 ± 0.120	1.455 ± 0.147
LVMI (g/kg)	3.532 ± 0.332	2.903 ± 0.258	3.405 ± 0.306
•EFS %	52.346 ± 2.847	56.116 ± 4.273	49.253 ± 6.436
•MFS %	30.362 ± 1.476	32.850 ± 3.008	26.710 ± 4.154
•PWSV (mm/s)	32.812 ± 1.692	33.867 ± 2.729	29.529 ± 1.821
EF %	0.882 ± 0.022	0.896 ± 0.029	0.824 ± 0.059
•Tei index	0.556 ± 0.042	0.561 ± 0.042	0.502 ± 0.017
Mitral E (cm/s)	106.778 ± 11.289	99.444 ± 12.721	93.875 ± 12.190
•Mitral A (cm/s)	49.000 ± 8.949	61.333 ± 11.438	36.375 ± 7.646
E/A	3.298 ± 0.819	2.795 ± 0.965	3.977 ± 1.060
•IVRT (ms)	23.222 ± 2.165	22.444 ± 1.796	21.125 ± 1.575

Heart rate (HR), left ventricular (LV) diastolic diameter (LVDD), left ventricular systolic diameter (LVSD), LV posterior wall thickness (PWT), LV septal wall thickness (SWT), left atrial diameter (LA), aortic diameter (AO), relative wall thickness (RWT), LV mass (LVM), LV mass index (LVMI), endocardial fractional shortening (EFS), midwall fractional shortening (MSF), posterior wall shortening velocity (PWSV), ejection fractional (EF), mitral E inflow (mitral E), mitral A (mitral A) wave, early (E)-to-late (A) diastolic mitral inflow (E/A) and isovolumetric relaxation time (IVRT). Aortic stenosis (AS), aortic stenosis treated with DEX (ASD) and aortic stenosis treated with ramipril (ASR). Results are presented as mean ± standard error of the mean. • means parametric test. Significance: ^+^ *p* < 0.05, vs. AS group and ^#^ *p* < 0.05, vs. ASD group. (^#^ This symbol is present in the group ASR for RWT parameter).

## Data Availability

The data presented in this study are available on request from the corresponding author.
